# Assessment of peritoneal microbial features and tumor marker levels as potential diagnostic tools for ovarian cancer

**DOI:** 10.1371/journal.pone.0227707

**Published:** 2020-01-09

**Authors:** Ruizhong Miao, Taylor C. Badger, Kathleen Groesch, Paula L. Diaz-Sylvester, Teresa Wilson, Allen Ghareeb, Jongjin Anne Martin, Melissa Cregger, Michael Welge, Colleen Bushell, Loretta Auvil, Ruoqing Zhu, Laurent Brard, Andrea Braundmeier-Fleming

**Affiliations:** 1 Department of Statistics, University of Virginia, Charlottesville, Virginia, United States of America; 2 Department of Medical Microbiology, Immunology and Cell Biology, SIU School of Medicine, Springfield, Illinois, United States of America; 3 Center for Clinical Research, SIU School of Medicine, Springfield, Illinois, United States of America; 4 Department of Obstetrics & Gynecology, SIU School of Medicine, Springfield, Illinois, United States of America; 5 Oak Ridge National Laboratory, Oak Ridge, Tennessee, United States of America; 6 Department of Ecology and Evolutionary Biology, University of Tennessee, Knoxville, Tennessee, United States of America; 7 National Center for Supercomputing Applications, University of Illinois at Urbana-Champaign, Champaign, Illinois, United States of America; 8 Applied Research Institute, University of Illinois at Urbana-Champaign, Champaign, Illinois, United States of America; 9 Department of Statistics, University of Illinois at Urbana-Champaign, Champaign, Illinois, United States of America; 10 Simmons Cancer Institute at SIU, Springfield, Illinois, United States of America; University of Insubria, ITALY

## Abstract

Epithelial ovarian cancer (OC) is the most deadly cancer of the female reproductive system. To date, there is no effective screening method for early detection of OC and current diagnostic armamentarium may include sonographic grading of the tumor and analyzing serum levels of tumor markers, Cancer Antigen 125 (CA-125) and Human epididymis protein 4 (HE4). Microorganisms (bacterial, archaeal, and fungal cells) residing in mucosal tissues including the gastrointestinal and urogenital tracts can be altered by different disease states, and these shifts in microbial dynamics may help to diagnose disease states. We hypothesized that the peritoneal microbial environment was altered in patients with OC and that inclusion of selected peritoneal microbial features with current clinical features into prediction analyses will improve detection accuracy of patients with OC. Blood and peritoneal fluid were collected from consented patients that had sonography confirmed adnexal masses and were being seen at SIU School of Medicine Simmons Cancer Institute. Blood was processed and serum HE4 and CA-125 were measured. Peritoneal fluid was collected at the time of surgery and processed for Next Generation Sequencing (NGS) using 16S V4 exon bacterial primers and bioinformatics analyses. We found that patients with OC had a unique peritoneal microbial profile compared to patients with a benign mass. Using ensemble modeling and machine learning pathways, we identified 18 microbial features that were highly specific to OC pathology. Prediction analyses confirmed that inclusion of microbial features with serum tumor marker levels and control features (patient age and BMI) improved diagnostic accuracy compared to currently used models. We conclude that OC pathogenesis alters the peritoneal microbial environment and that these unique microbial features are important for accurate diagnosis of OC. Our study warrants further analyses of the importance of microbial features in regards to oncological diagnostics and possible prognostic and interventional medicine.

## Introduction

Epithelial ovarian cancer (OC) is the most deadly cancer of the female reproductive system. In the US alone, over 22,530 new cases and 13,980 deaths were estimated for 2019 [1]. To date, there is no effective screening method for early detection of OC [2]. Consequently, greater than 60% of OC cases are diagnosed at an advanced stage (III, IV) [1]. Because women with early stage OC normally report non-specific symptoms (bloating, upset stomach, constipation), workup is usually delayed until a pelvic mass is found during a routine annual exam or found incidentally during abdominal imaging [2, 3]. When an adnexal mass is discovered, the current diagnostic armamentarium may include ultrasound assessment of the tumor and analyses for serum levels of tumor markers, Cancer Antigen 125 (CA-125) and Human epididymis protein 4 (HE4) [2, 4, 5]. Recently, the American College of Radiology Ovarian-Adnexal Reporting Data System Committee has standardized the terminology to be used by sonographers, to reduce the “subjectivity” of ultrasound assessment for ovarian cancer diagnosis [6]; however, this review concluded that multimodal approach of sonography and elevated CA-125 may be most beneficial. The error with using imaging (even if standardized) and CA-125 is that these modalities are still flawed, not standard practice in every oncology clinic, and are not effective for early diagnosis of disease [7, 8]. CA-125 is elevated in most cases of epithelial OC (~80%) [9–11]; however, CA-125 is also known to be elevated in patients with benign tumors [3] and a variety of other health conditions such as endometriosis, uterine fibroids, diverticulitis and cirrhosis of the liver [10, 12–14]. Additionally, elevated levels of CA-125 (≥ 35 U/mL for postmenopausal women) may be found in only ~50% of women with stage I OC [10, 14]. Thus, the literature suggests the use of more than one biomarker as a means of increasing the sensitivity for the early detection of OC [10, 14, 15]. HE4 is a relatively new biomarker that is overexpressed by ovarian carcinomas [10, 11, 16]. HE4 has been reported to be the most sensitive biomarker for detecting stage I OC and it is expressed by approximately 30% of OC cases that do not express CA-125 [3]. Furthermore, the U.S. Preventative Task Force recommends against screening for OC in low-risk, asymptomatic population with the current tools available [17]. Thus, it is essential to develop new tools to identify women at risk and accurately diagnose prior to surgical intervention, which ultimately will help maximize successful outcomes through personalized medicine.

An innovative approach to diagnose different cancer phenotypes is through profiling a patient’s microbial features (known as the microbiome). Previous studies have shown that microorganisms (bacterial, archaeal, and fungal cells) residing in mucosal tissues including the gastrointestinal and urogenital tracts can be altered by varied disease states, and these shifts in microbial dynamics may help to diagnose disease states before they become symptomatic [18–22]. An individual’s inflammatory status is also known to impact microbial dynamics and patients who have immune mediated diseases have unique microbial profiles [23–26]. The known association between OC and peripheral inflammation suggests the likelihood of a unique microbial signature associated with OC. Recently, through Next Generation Sequencing (NGS), research has shown that human microbial profiles are associated with cancer development and progression [27, 28]. In addition, human associated microbiota have the potential to alter estrogen metabolism through enzymatic cleavage of glucoronidases that shunt estrogens for excretion or absorption. Through this process, microbial profiles have the potential to either suppress or promote estrogen driven cancers [27–29], which may explain the variability in response to endocrine treatments between individuals. However, this has only been reported through analysis of vaginal or gastrointestinal microbial profiles. In this work, we examined the microbial profile of peritoneal fluid, due to its intimate relationship with the ovarian tumor microenvironment. First we used machine learning analyses to select the top microbial features. We then combined our selected microbial feature set with CA-125 and HE4 serum and peritoneal fluid levels to determine if these combined feature sets may be a superior predictor of disease using standard Area Under the Curve (AUC) and Receiver Operating Curve (ROC) performance measures.

**Fig 1:**
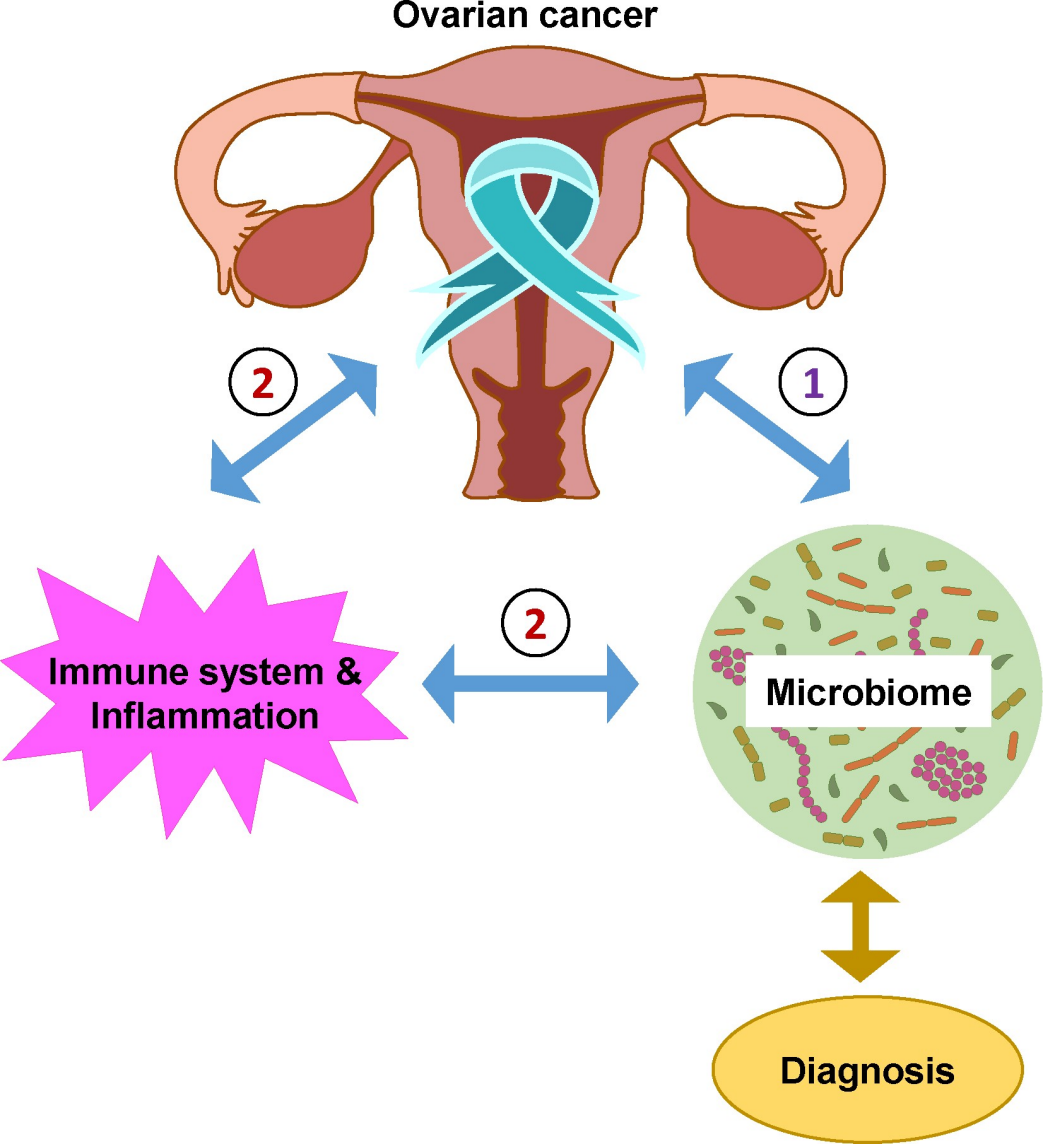
Working hypothesis and model. The diagram illustrates how cancer pathophysiology may alter the microbial features within the tumor microenvironment. These alterations may be: 1) directly through the production of metabolites by malignant cells or 2) through the alteration of the patient’s immune system/inflammatory status. We hypothesize that changes in microbial profiles associated with the presence of ovarian cancer may be characterized and used as diagnostic tools.

We hypothesized that the altered microbial environment induced by the onset of ovarian cancer pathogenesis will alter microbial dynamics (**Fig 1**). These changes to microbial profiles and site specific analysis of tumor markers, which can be easily and rapidly identified using existing toolsets, could then serve as a more sensitive and specific platform for diagnosis and potential staging of OC, rather than analysis of serum biomarkers and ultrasound imaging either alone or together. This biological systems approach along with machine learning analysis would, therefore, ultimately represent a rapid, user-friendly, reliable, and point-of-care tool that could enhance diagnostic accuracy for OC and would enable the development of decision trees (surgical and pharmaceutical) to improve oncologic care for OC. The discovery of a potential “onco-biome” has implications for determining prognosis, staging and even novel screening tests utilizing microbial sampling.

## Materials and methods

### Patient eligibility and recruitment process

This was a cross-sectional pilot study approved by the local Institutional Review Board (Springfield Committee for Research Involving Human Subjects) under the protocol 12–656. The experimental group (n = 30) consists of eligible patients (age ≥ 30) who presented to the Department of Obstetrics & Gynecology, Gynecological Oncology division at Southern Illinois University School of Medicine for surgical management of an adnexal mass or suspected ovarian carcinoma. These patients were scheduled for an oophorectomy, bilateral salpingo-oophorectomy (BSO), hysterectomy, hysterectomy/BSO, staging and/or debulking via laparotomy or laparoscopy. Patients with a previously diagnosed malignancy within the pelvis or abdomen (including recurring or previously diagnosed OC patients) were excluded; however, as expected from previous clinical experience and reported data [1], most of the eligible patients who were subsequently diagnosed with OC had advanced OC (stages III and IV). Eligible patients were invited to participate in this study when they presented to the Gynecology Oncology clinic for preoperative evaluation and diagnostic workup. Informed consent was obtained at this preoperative visit. Although the number of samples used in this study is relatively low, we incorporated robust statistical procedures including leave-one-out cross-validation [30]. These methods are specifically designed for low sample number and would effectively prevent overfitting. Hence, the discoveries and conclusions of our analysis are statistically sound.

### Sample collection schedule and procedures

We obtained peritoneal fluid at the time of surgery from peritoneal washings that were performed per standard of care. When present, ascites was first aspirated and then a standardized volume (300 mL) of isotonic saline (0.9% NaCl) was infused into the peritoneal cavity and then re-aspirated to serve as our collection sample. Once collected, the fluid was refrigerated until further processing (detailed below) was complete.

### CA-125 and HE4 tumor marker levels

Serum tumor marker levels were measured preoperatively as standard of care and this information was extracted from the subject’s electronic medical record.

Final pathology results from ovarian tissue sample(s) were utilized as the reference standard for diagnosis of OC. Peritoneal fluid was centrifuged at 1300 x g for 20 min., supernatant was removed and aliquots stored at -80°C until analysis of CA-125 (Siemens Healthineers Immulite 2000; Tarrytown, NY) and HE4 (EIA kit Fujirebio Diagnostics, Inc.; Malvern, PA) was performed at the Division of Medical Screening and Special Testing Reference laboratory (Women and Infants’ Hospital Providence, RI). For serum, standard laboratory reference ranges were defined as < 35 U/mL for a normal CA-125 and < 140 pmol/L for a normal HE4, absolute values were used for measurement of performance. For peritoneal fluid levels, > 500 for CA-125 (U/mL) and HE4 (pmol/L) were used which was more conservative than those previously published [31, 32] to increase the sensitivity of their performance. Due to the variability of peritoneal fluid tumor marker levels, we classified patients based on our stated cut-off values for measurement of performance. The peritoneal fluid pellet was used for DNA isolation and sequencing of microbial organisms.

### Next generation sequencing (NGS) of microbial communities

Next Generation Sequencing (NGS) of the bacterial 16S RNA gene was performed to characterize each subject’s individual peritoneal microbial signature. DNA extraction of samples was performed using the PowerSoil DNA isolation kit (Qiagen). Amplicon sequencing targeting the V4 region of the 16S rRNA gene was employed on DNA extracted from each sample. Initial microbial sequence data processing was carried out using a combined USEARCH [33] and QIIME [34] pipeline. Briefly, fastq files were loaded onto a server, where they were processed (demultiplexed, trimmed, filtered, operational taxonomic unit (OTUs) clustered, removal of chimeric sequences, and taxonomy assigned via RDP) and then mapped using USEARCH at 97% sequence identity threshold. The resulting OTU table was then parsed to obtain the different OTU counts per sample and finally combined with taxonomy information and other metadata into a BIOM-formatted file [35].

Once the BIOM-formatted file was obtained, community analyses were conducted using the VEGAN [36] and phyloseq packages [37] in R [38]. Additional analyses were conducted using QIIME [39] and GALAXY software packages [40–42].

### Microbial feature selection: Machine learning analyses

**Fig 2:**
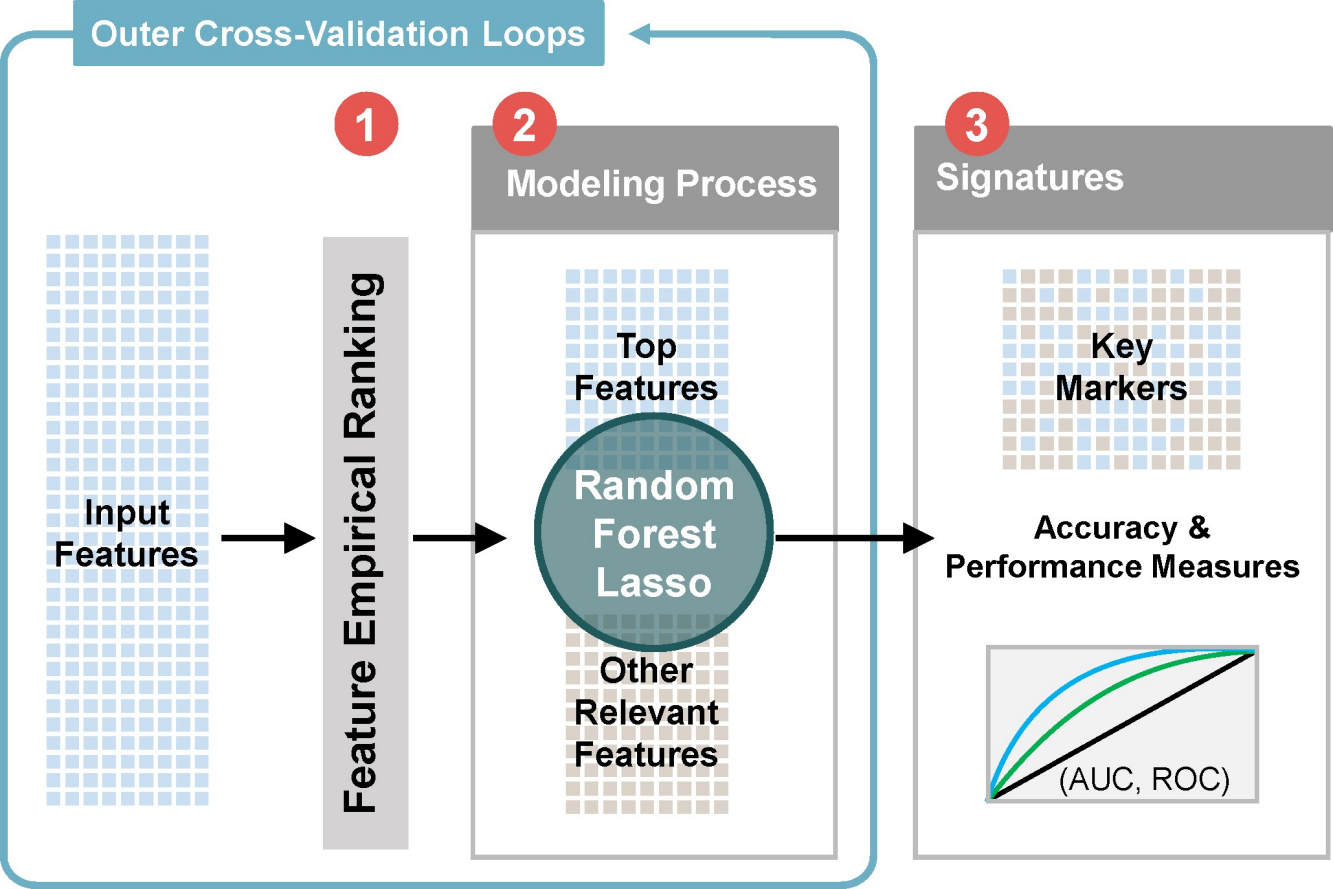
Feature selection and predictive modeling. The flow chart summarizes our analytical processes of data analysis. Feature empirical ranking was utilized to select subsets of top and relevant features which are then utilized in the outer cross-validation loop of the Integrative Modeling Process. Ultimately, signature profiles were identified and predictive accuracy and performance of the models were evaluated.

To identify peritoneal microbial features associated with OC, we utilized an ensemble methodology which combines traditional statistical approaches with machine learning methods to perform key predictor selection and ranking. The feature selection analysis is outlined in **Fig 2**. We employed a two-stage feature screening (as part of the flowchart in **Fig 2**): First, OTUs are assessed through ensemble feature selection. The ensemble feature selection includes random forest variable importance [43], Lasso coefficient [44], t-test, distance correlation [45], and Mann-Whitney test [46]. A review of these model based approaches can be found in Zhou & Gallins, 2019 [47]. After selecting a subset of features that contains significant OTUs, we rank them based on the Mann-Whitney test [46], which is believed to be more robust. In our analysis, we ran random forest and Lasso using both the top-18 and top-43 OTUs, and used leave-one-out cross-validation [48, 49] using Lasso [50] and random forests [51] to compare the two choices. Cross-validation is a statistical out-of-sampling-testing procedure that can be utilized to estimate the prediction error. Details regarding the next stage of feature selection will be introduced in the results. Note that we did not choose to fit a model to all OTUs but rather selected the significant/important ones because either random forest or Lasso provided a valid p-value, also because we wanted to ensure that we did not overfit our model due to the small sample size. For our data, the top OTU model yields smaller error for both Lasso and random forests, hence it was used for the final result, which compared their predictive values with existing clinical measurements.

### Statistical modeling of the ovarian cancer classification

#### Comparing OTUs with existing clinical measurements

We considered two sets of models to assess the predictive power of OTUs (with phylogenetic grouping). In the first set, we considered two different baseline models using either the serum features or the peritoneal fluid features as predictors in the classification. Age and BMI serve as control features. The first baseline model was defined as serum (S) tumor marker levels (CA-125 and HE4) with Age and BMI. The second baseline model was defined as peritoneal fluid (PF) tumor marker levels (CA-125 and HE4) with Age and BMI. The comparison models utilized different combinations of OTU features, along with Age and BMI with or without tumor marker values. From our previous feature selection approach, we selected the top ranked OTUs for this modeling step which reduces the total number of potential features.

#### Modeling approaches

Due to the small sample size, there was a high risk of overfitting. Hence, we considered two approaches, random forests and logistic regression with Lasso penalty [50]. Random forests [51] are ensemble classifiers consisting of a large number of classification trees. It is among the state-of-the-art machine learning tools and is known to perform well for small sample-size studies. The Lasso logistic regression model [52] approach is included for comparison due to its simple model structure and easy interpretation. This is a penalized linear model, which imposes an L1 penalty on the coefficients. The L1 penalty shrinks some of the coefficients to exactly 0, which effectively removes irrelevant features to better deal with small sample size.

#### Cross-validation and evaluation metric

We used Leave-one-out cross-validation [48] to evaluate and compare the performance of our models. The Leave-one-out cross-validation works in the following way: First, we selected one subject out of the N total samples as the testing sample. Then the rest N-1 samples are used to fit a classification model. The model is then evaluated on the testing sample to assess the performance. We then rotate this procedure to all N possible testing samples and calculate the overall classification error by averaging all the results.

The random forest classifier consists of an ensemble of trees. Given a new sample, each of these trees will produce one classification label. We can view the proportions of each label as probabilities of the sample being in that class. Therefore, for each testing subject in the cross-validation, the probability of that subject having ovarian cancer can be calculated. The subject can be predicted as having OC if that probability is above 50%. However, noticing that our dataset was slightly unbalanced in the response label, we used the receiver operating characteristic (ROC) curve and calculated the area under the ROC curve (AUC) as the measure of performance. This is a more robust approach compared to simple classification errors.

#### Tuning parameter and model selection of machine learning models

We use the “cv.glmnet” function in R package “glmnet” for Lasso [53], and the “randomForest” function in R package “randomForest” [54] for random forest classifier.

The penalty parameter of Lasso is determined through 10-fold cross validation. Specifically, during each step of the aforementioned leave-one-out cross validation, for a set of candidate penalty parameter values, we calculate the classification error for each candidate value through 10-fold cross validation on the N -1 training samples. We then choose the parameter value that yields the smallest classification error, fix the penalty parameter at that value, and fit the model using all N -1 training samples.

For random forest classifier, there are also more than one tuning parameters. However, due to the high complexity of random forest and relatively small sample size, fine tuning of its parameters may increase the risk of overfitting. Therefore, we only use the default parameter values provided by the R package “randomForest”. It turns out this set of parameter values already leads to accurate classification results.

## Results

### Study population

A total of 44 subjects were enrolled in this study. After withdrawing subjects due to retracted consent, screen fail, discovery that malignant masses originated in organs other than the ovaries and presence of ovarian borderline tumors, we analyzed samples from 30 subjects. Of these, 10 were diagnosed with OC and 20 had benign ovarian masses. The majority of the subjects in the ovarian cancer cohort of this study had advanced disease (6 patients with stage III, 2 stage IV and 2 stage II). Most of these patients had serous OC (9 subjects), there was 1 patient diagnosed with grade 2 endometrioid OC. In the benign masses cohort, the vast majority of the subjects were diagnosed with either cystadenomas (9 subjects) or cystadenofibromas (6 subjects), while 1 subject had a cystadenofibroma in one ovary and a cystadenoma in the other, 2 subjects had hemorrhagic corpus luteum cysts, 1 subject had cystic endometriosis and 1 subject had a mature cystic teratoma.

Demographic information and menopausal status are shown in **Table 1**. We found that patient age and BMI were significant factors in determination of malignancy or benign adnexal tumors. Therefore, these 2 factors were included in all further downstream statistical models. Median values for tumor markers found in serum and peritoneal fluid are summarized in **Table 1**.

**Table 1:** Study population’s demographic information and tumor marker levels. Tumor markers values were compared using non-parametric Mann-Whitney test. All other numerical variables were compared through Student’s t-test.

		Benign (n = 20)	Malignant (n = 10)	*p*-value
**Age (Mean ± S.E.M.)**		56.0 ± 3.1	66.1 ± 3.0	0.047
**Race**	**Caucasian**	20	9	
	**African American**	0	1	
**BMI (Mean ± S.E.M.)**		36.4 ± 2.2	30.3 ± 1.9	0.048
**Menopausal status**	**Pre-menopausal**	4	0	
	**Post-menopausal**	16	10	
**Serum levels (Median)**	**CA-125 (U/ml)**	19.5	362	0.0003*
	**HE4 (pM)**	58	265	0.0008*
**Peritoneal levels (Median)**	**CA-125 (U/ml)**	74	3032	0.0339*
	**HE4 (pM)**	68.5	6530	0.0013*

*p < .05

### Microbial DNA in peritoneal fluid

**Fig 3:**
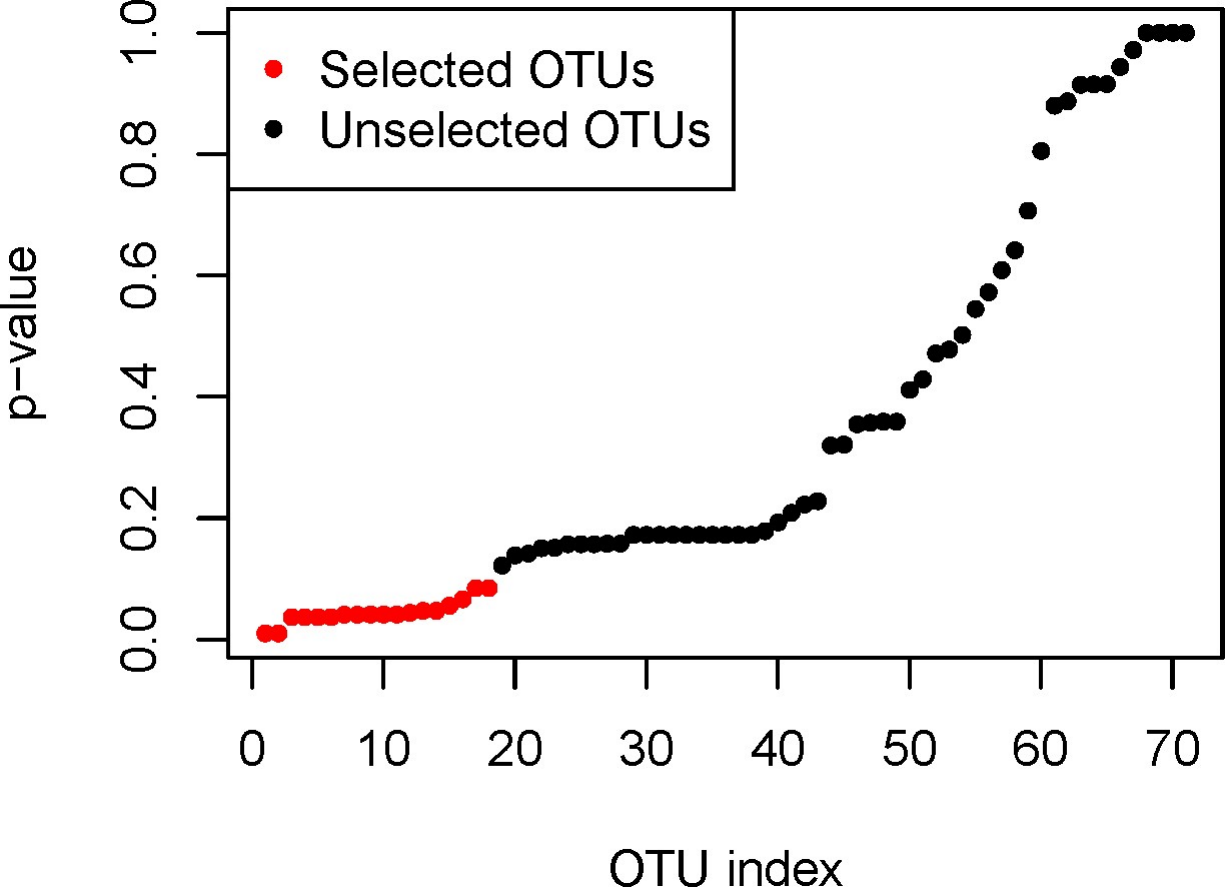
Microbiome: Principal Coordinates Analysis (PCoA). Non-metric dimensional analysis of 16S microbial features in peritoneal fluid from patients with adnexal masses shows distinctive clustering of sample points from patients diagnosed with OC (red dots, n = 10/30) compared to those with benign masses (blue dots, n = 20/30).

The principal coordinates analysis (PCoA) plot shown in **Fig 3** provides graphic visualization of clustering of OTUs identified via sequencing of the 16S microbial DNA in the peritoneal fluid from all study subjects. We observed, with the exception of one outlier, a high level of clustering of the data points from individuals with confirmed OC (**Fig 3**, red dots; n = 10/30). This result was indicative of the presence of similar bacterial community profiles within the peritoneal fluid samples from subjects with OC. However, patients diagnosed with benign masses exhibited a more diverse profile of bacterial communities evidenced by the higher dispersion in the distribution of the data points from individuals with benign masses (**Fig 3**, blue dots; n = 20/30).

### Machine based learning: Microbial Feature Selection

**Fig 4:**
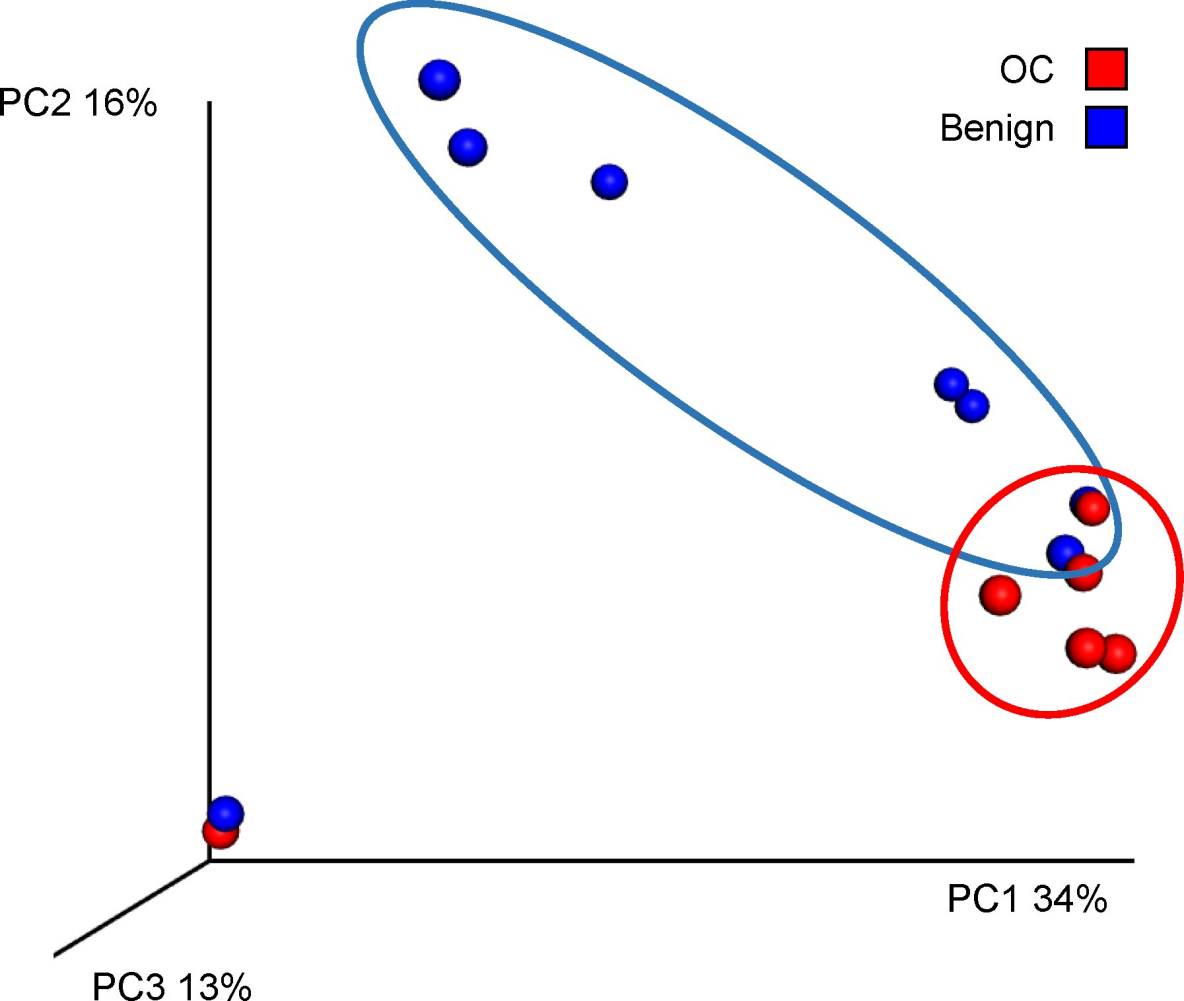
Microbial Feature Selection. The plot shows screening of the top 76 features by p-value ranking. Each dot represents one feature. The 18 microbial features marked in red are highly associated with patients diagnosed with OC.

The potential of our peritoneal microbial feature set to predict the outcome measure of OC was tested using the methodology described previously and depicted in **Fig 2.** All identified microbial features were empirically ranked by their presence and abundance in the peritoneal fluid of patients with pathologically confirmed OC. The top 76 OTUs were selected after performing an ensemble screening which tested the importance of each microbial feature by analyses of 10 different algorithms both marginal (Dcor, Mann-Whitney, MIC and t-test) and embedded (Boruta, ElasticNet, Lasso, Random Forest, Randomized Lasso and SVM). Following this initial selection, we then performed a whole screening step on the top 76 features by p-value ranking. In **Fig 4**, we plotted the obtained p-values in increasing order. There are two large gaps in **Fig 4**, corresponding to top-18 and top-43 OTUs respectively. These two gaps suggest a natural cut-off at the top-18 and top-43 OTUs for the final model. As a result, 18 different microbial features were identified as being highly associated with patients with OC (**Fig 4 and Table 2**).

**Table 2:** List of the top 18 microbial features highly specific to OC pathology.

OTU #	Taxonomy	Function	Ref.
OTU_7	Bacteroidetes; Bacteroidia; Bacteroidales; Prevotellaceae; Prevotella; stercorea	Human feces or vagina. Exact role unknown but Prevotella species are known to have pathogenic function in inflammatory diseases.	[59–61]
OTU_113	Bacteroidetes; Bacteroidia; Bacteroidales; Rikenellaceae	Fecal microbiota that is associated with ESR1 function. Also associated with inflammation induced by high fat diet.	[62, 63]
OTU_157	Bacteroidetes; Bacteroidia; Bacteroidales; Prevotellaceae; Prevotella	Human feces or vagina. Exact role unknown but Prevotella species are known to have pathogenic function in inflammatory diseases.	[59–61]
OTU_233	Bacteroidetes; Bacteroidia; Bacteroidales; [Odoribacteraceae]; Odoribacter	Fecal microbiota. Some species associated with abdominal abscess and loss of these species lead to host inflammation.	[64, 65]
OTU_1388	Bacteroidetes; Bacteroidia; Bacteroidales; Bacteroidaceae; Bacteroides; ovatus	Fecal microbiota. Negatively associated with acute GVHD.	[66]
OTU_158	Firmicutes; Clostridia; Clostridiales; Lachnospiraceae	Fecal Microbiota. Increased in non-alcoholic fatty liver disease but decreased in cirrhotic liver disorders	[67]
OTU_284	Firmicutes; Clostridia; Clostridiales; Lachnospiraceae; Roseburia	Fecal Microbiota that produces short chain fatty acids. Reduced in IBD.	[68]
OTU_297	Firmicutes; Clostridia; Clostridiales; Ruminococcaceae; Oscillospira	Fecal Microbiota increased with Chron’s disease	[69]
OTU_770	Firmicutes; Clostridia; Clostridiales; Lachnospiraceae	Fecal Microbiota. Increased in non-alcoholic fatty liver disease but decreased in cirrhotic liver disorders	[67]
OTU_884	Firmicutes; Clostridia; Clostridiales; Lachnospiraceae; Clostridium; colinum	Unknown	
OTU_914	Firmicutes; Erysipelotrichi; Erysipelotrichales; Erysipelotrichaceae; [Eubacterium]; dolichum	Gut Microbiota. Highly immunogenic and associated with inflammatory gastrointestinal disorders.	[70]
OTU_1268	Firmicutes; Clostridia; Clostridiales	Gut and vaginal microbiota. Many species of Clostridia have pathogenic potential.	[71]
OTU_1836	Firmicutes; Clostridia; Clostridiales; Ruminococcaceae; Faecalibacterium	Gut Microbiota. Decrease is associated with IBS	[72]
OTU_217	Proteobacteria; Alphaproteobacteria; RF32	Highly diverse class. Pathogenic capability may be through an increase vascular permeability and vascular damage.	[73]
OTU_570	Proteobacteria; Betaproteobacteria; Burkholderiales; Alcaligenaceae; Sutterella	Gut microbiota. Associated with nonalcoholic steatohepatitis.	[74]
OTU_1092	Proteobacteria; Alphaproteobacteria; Rhizobiales; Bradyrhizobiaceae; Bradyrhizobium	Plant bacterium that are nitrogen fixers.	[75]
OTU_1195	Proteobacteria; Alphaproteobacteria; Rickettsiales; mitochondria	Highly diverse class. Pathogenic capability may be through an increase vascular permeability and vascular damage.	[73]
OTU_274	Verrucomicrobia; Verrucomicrobiae; Verrucomicrobiales; Verrucomicrobiaceae; Akkermansia; muciniphila	Gut microbiota. Has anti-inflammatory effects and may increase immunotherapy effectiveness in cancer patients.	[76, 77]

Of the microbial features identified, the common origin was that of microbiota found in human gut or feces. The finding of these species in the peritoneal cavity can be through the migration of these species from the colon to the urogenital canal. It may also be that the pathogenesis of OC causes intestinal permeability leading to translocation of these species into the peritoneal cavity, however the exact mechanism is unknown and was not the focus of this investigation. The functionality of the microbial features varied but was centralized around inflammatory mediators. It is widely known that the pathogenesis of OC induces peritoneal inflammation and that this is one possible mechanism for disease metastasis [55–58]. Of particular interest, we found 3 different microbial features that may be directly related to disease pathogenesis; estrogen responsiveness (Rikenellaceae (order) of the Bacteroidetes (Phylum)), vascular permeability (Alphaproteobacteria (Class) of the Proteobacteria (Phylum)) and anti-inflammatory properties (*Akkermansia*; *muciniphila* of the Verrucomicrobia (Phylum)). Further analysis of our entire selected microbial feature list needs to be completed and may identify potential targets for therapeutic intervention.

### Predictive analysis: Diagnostic accuracy

To determine which biological features are important for diagnostic accuracy, we tested the ability of our microbial feature selection to diagnose OC and compared it against the currently used biological markers (age, BMI, serum CA-125/HE4 tumor marker levels) and also CA-125/HE4 tumor marker levels from the peritoneal fluid as our baseline models. Four subjects with benign diagnosis and two subjects with confirmed OC were excluded from this predictive analysis due to lack of pre-operative serum HE4 testing.

**Fig 5:**
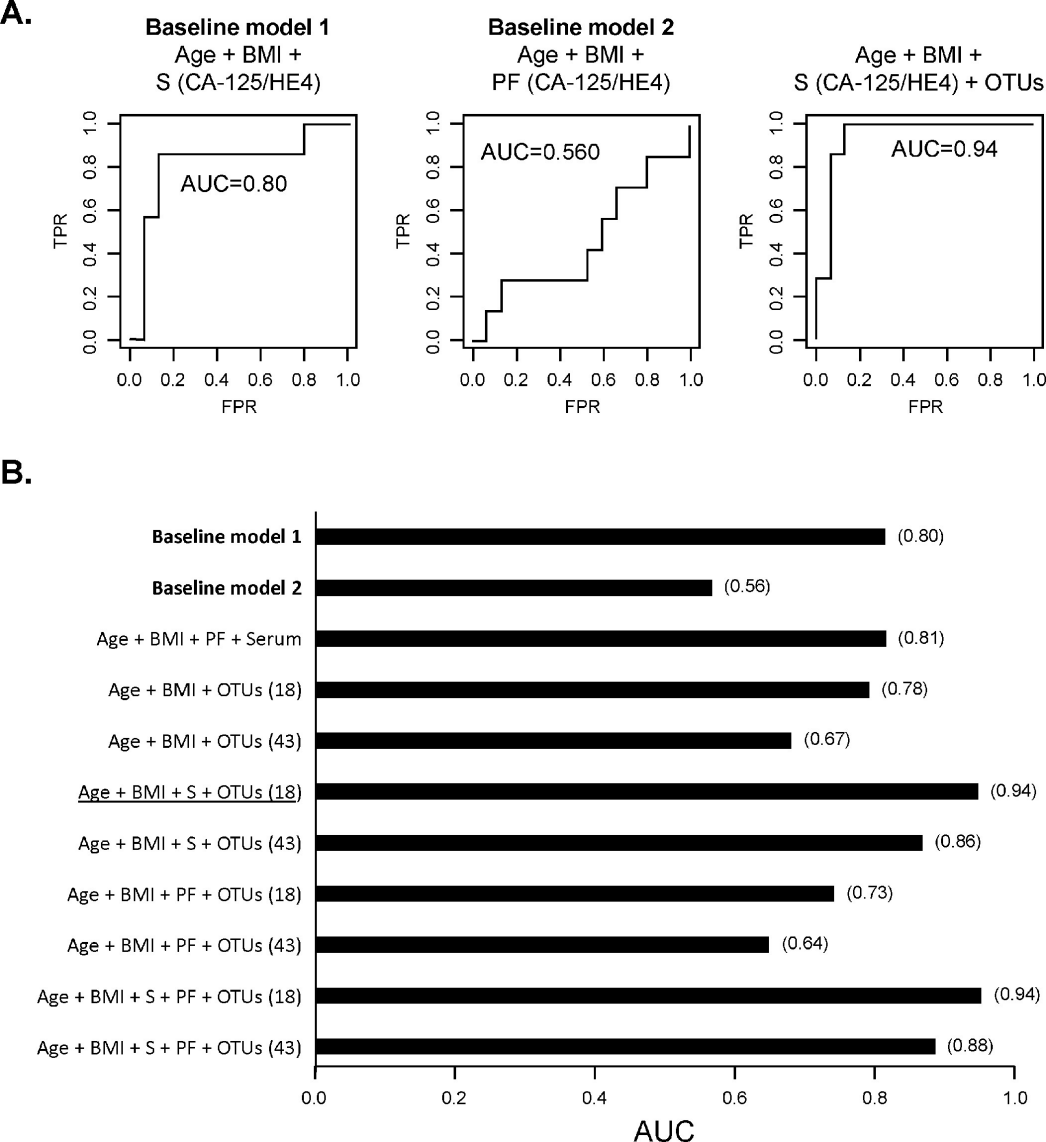
AUC Analysis of models for OC diagnostic potential. **A.** AUC calculations for the baseline models 1 and 2 (left and middle panels, respectively) and after adding the 18 top ranked OTUs to baseline model 1 (right panel). **B.** Summary of AUC values obtained for baseline models and various feature combinations (S: serum; PF: peritoneal fluid).

Initially, we measured the AUC for our baseline models using random forest analyses. Measures of CA-125 and HE4 levels from serum, patient age and BMI (baseline model 1) had a 0.804 AUC performance compared to 0.560 for peritoneal fluid CA-125/HE4, patient age and BMI (**Fig 5A**). Combination of serum and peritoneal CA-125/HE4 data, together with patient age and BMI did not significantly change AUC performance (0.806) (**Fig 5B**). This indicated that tumor marker levels from the peritoneal fluid did not enhance the predictive power of current clinical diagnostic tests (baseline model 1).

We then determined the value of adding microbial features to our model fitting. We found that the addition of the selected 18 OTUs with baseline model 1 had the best performing model in AUC (0.94) of OC detection (**Fig 5A**). This model decreased in performance when we used 43 microbial OTU features (0.857) (**Fig 5B**).

Variations of feature combinations were analyzed and included in the predictive model, including OTUs with patient age and BMI (AUC = 0.782), OTUs with peritoneal fluid tumor marker levels (AUC = 0.732), and OTUs with tumor marker levels from serum and peritoneal fluid (AUC = 0.940). In each feature combination, as we increased the OTU feature set from the 18 highly selected variables to 43 variables, we found a decrease in AUC performance **(Fig 5B)**.

Hence, our results suggest that, with age and BMI as control features, the serum features outperform peritoneal tumor marker level features; however, microbial features within the peritoneal fluid enhance the performance measurement of serum tumor marker levels for OC diagnosis.

## Discussion

In this study, the diagnostic utility of peritoneal microbial profiling and tumor marker (CA-125/HE4) levels in the peritoneal fluid was tested for prediction of OC. A machine learning approach was utilized to perform predictive analysis of different datasets and compare the accuracy of currently utilized diagnostic tools (serum tumor marker levels) against that of our potential novel biomarkers (peritoneal tumor marker levels and microbial profiles). Our results indicated that individuals diagnosed with OC have unique peritoneal microbial profiles which are similar amongst OC patients, but have distinct characteristics compared to those from subjects with benign adnexal masses. Indeed, we found that the best performing model for prediction of malignancy (diagnosis) is the one that combines analysis of peritoneal OTUs and serum tumor marker levels.

We initially hypothesized that measuring cancer markers in the vicinity of the pelvic mass would have an increased diagnostic value compared to the standard of care measurements performed in serum. However, analysis of diagnostic performance by AUC, indicated that tumor marker levels measured in serum were better predictors of malignancy than those measured in the peritoneal fluid. Our principal coordinates analysis indicated that not only the existence of a unique microbial signature in subjects diagnosed with OC exists, evidenced by the fact their samples clustered separately from samples obtained from subjects with benign adnexal masses, but also suggested that the presence of disease produces a decrease in microbial diversity (less dispersion in samples of OC vs. benign subjects). This loss of diversity associated with OC has been previously described in cancer patients [78–81] along with the generally accepted idea that more diverse microbiomes are more robust and healthier than less diverse ones [82].

Feature selection analysis identified 43 unique microbial features present in all samples from subjects diagnosed with OC. Interestingly, 18 of these microbial features were identified through our unique feature selection approach to be highly important in OC subjects. Further descriptive analyses, such as metagenomic or transcriptomic profiling of these microbial features would potentially identify microbial features that are involved with the pathophysiology of OC or that could potentially be used for OC staging and screening.

Together, the principal coordinates and feature selection analyses support the peritoneal microbiome as an important diagnostic tool in OC. Indeed, predictive analysis of diagnostic accuracy performed via machine learning strategies indicated that adding microbial data to the serum cancer marker level model improved overall diagnostic performance. However, a predictive model including microbial data alone had reduced accuracy, indicating the need to utilize serum cancer markers in combination with OTUs for optimal results.

The machine learning approach has a broad variety of applications ranging from speech recognition to financial market analysis and it is becoming an increasingly important part of bioinformatics. More recently, the use of this approach to analyze microbiome data in order to predict disease and, specifically, cancer diagnoses has been reported [83, 84], but to date no data has been published regarding its utility in the diagnosis/prognosis of OC.

We are aware that the samples analyzed in this work were collected during surgery from symptomatic patients. Part of the standard surgical management of patients with adnexal masses, involves collecting peritoneal fluid to assess the presence of malignant cells, which can also be used to determine its microbiome’s potential diagnostic value. However, peritoneal fluid with the same characteristics and composition as that collected during surgery can also be obtained without surgery via a procedure called culdocentesis. This procedure allows for peritoneal fluid to be collected from the cul-de-sac (pouch of Douglas) of the female patient. A spinal needle is introduced through the posterior vaginal fornix into the peritoneal space of the pouch of Douglas to allow for aspiration of peritoneal fluid [85]. Culdocentesis could potentially be used for screening, as it can be performed in an outpatient setting with local anesthesia, reducing costs and potential morbidity in comparison with surgery; in fact there is a large body of evidence supporting culdocentesis’ safety [86–90]. In summary, we evaluated the ability of the microbial profile from the peritoneal fluid to identify patients with OC before testing the practical implementation of this potential tool. As mentioned above, in OC patients symptoms typically arise at advanced stages, which leads to poor survival. However, the current recommendations are against screening OC in asymptomatic women with no family history of the disease mainly due to the poor specificity and elevated number of false positives obtained with the current diagnostic tools [17]. Developing a minimally invasive, highly accurate screening test with a minimal number of false positives would be critical for: earlier diagnosis, improved patient survival and to guide decision trees for surgical management of disease or benign adnexal masses. If peritoneal microbial feature analysis determines that a patient has early stage OC then the surgeon could proceed with minimally invasive approaches, such as robotically assisted laparoscopy, to safely excise the primary tumor and stage the cancer; thus, improving patient recovery time and quality of life [91, 92]. In this regard, the urogenital and gastrointestinal microbial profiles (from samples obtained non-invasively) are currently being tested utilizing this novel approach. This would potentially enable patients to be effectively screened at various significant ages to determine if further workup is needed prior to symptoms being noticed.

In summary, the application of microbial analysis and machine learning approaches for diagnosis and prognosis of OC is a promising robust tool that has the potential of improving health outcomes in this devastating disease and positively impacting gynecologic oncologic care. Our pilot investigation into identification of microbial features that are associated with OC which may have diagnostic potential warrants further investigations on the benefits of adding peritoneal fluid microbial profiling to existing tool sets for improving early detection and development of effective treatments of OC.

## References

[1] Cancer Stat Facts: Ovarian Cancer: National Cancer Institute; 2019 [3/4/2019]. Available from: https://seer.cancer.gov/statfacts/html/ovary.html.

[2] Duffy MJ. Use of Biomarkers in Screening for Cancer. Advances in experimental medicine and biology. 2015;867:27–39. 10.1007/978-94-017-7215-0_3 . 26530358

[3] Andersen MR, Goff BA, Lowe KA, Scholler N, Bergan L, Drescher CW, et al Use of a Symptom Index, CA125, and HE4 to predict ovarian cancer. Gynecol Oncol. 2010;116(3):378–83. 10.1016/j.ygyno.2009.10.087 19945742 PMC2822097

[4] Granato T, Porpora MG, Longo F, Angeloni A, Manganaro L, Anastasi E. HE4 in the differential diagnosis of ovarian masses. Clin Chim Acta. 2015;446:147–55. 10.1016/j.cca.2015.03.047 . 25892674

[5] ACOG Practice Bulletin. Management of adnexal masses. Obstet Gynecol. 2007;110(1):201–14. Epub 2007/07/03. 10.1097/01.AOG.0000263913.92942.40 . 17601923

[6] Shetty M. Imaging and Differential Diagnosis of Ovarian Cancer. Semin Ultrasound CT MR. 2019;40(4):302–18. Epub 2019/08/04. 10.1053/j.sult.2019.04.002 . 31375171

[7] Giampaolino P, Della Corte L, Foreste V, Vitale SG, Chiofalo B, Cianci S, et al Unraveling a difficult diagnosis: the tricks for early recognition of ovarian cancer. Minerva Med. 2019;110(4):279–91. Epub 2019/05/14. 10.23736/S0026-4806.19.06086-5 . 31081307

[8] Kamal R, Hamed S, Mansour S, Mounir Y, Abdel Sallam S. Ovarian cancer screening-ultrasound; impact on ovarian cancer mortality. Br J Radiol. 2018;91(1090):20170571 Epub 2018/08/14. 10.1259/bjr.20170571 30102555 PMC6350495

[9] Bottoni P, Scatena R. The Role of CA 125 as Tumor Marker: Biochemical and Clinical Aspects. Advances in experimental medicine and biology. 2015;867:229–44. 10.1007/978-94-017-7215-0_14 . 26530369

[10] Nossov V, Amneus M, Su F, Lang J, Janco JM, Reddy ST, et al The early detection of ovarian cancer: from traditional methods to proteomics. Can we really do better than serum CA-125? Am J Obstet Gynecol. 2008;199(3):215–23. 10.1016/j.ajog.2008.04.009 . 18468571

[11] Van Gorp T, Cadron I, Despierre E, Daemen A, Leunen K, Amant F, et al HE4 and CA125 as a diagnostic test in ovarian cancer: prospective validation of the Risk of Ovarian Malignancy Algorithm. British journal of cancer. 2011;104(5):863–70. Epub 2011/02/10. 10.1038/sj.bjc.6606092 21304524 PMC3048204

[12] Szubert M, Suzin J, Wierzbowski T, Kowalczyk-Amico K. CA-125 concentration in serum and peritoneal fluid in patients with endometriosis—preliminary results. Arch Med Sci. 2012;8(3):504–8. 10.5114/aoms.2012.29407 22852007 PMC3400917

[13] Nisenblat V, Bossuyt PM, Shaikh R, Farquhar C, Jordan V, Scheffers CS, et al Blood biomarkers for the non-invasive diagnosis of endometriosis. The Cochrane database of systematic reviews. 2016;(5):CD012179 10.1002/14651858.CD012179 . 27132058 PMC7076288

[14] Moore RG, Brown AK, Miller MC, Skates S, Allard WJ, Verch T, et al The use of multiple novel tumor biomarkers for the detection of ovarian carcinoma in patients with a pelvic mass. Gynecol Oncol. 2008;108(2):402–8. 10.1016/j.ygyno.2007.10.017 . 18061248

[15] Partheen K, Kristjansdottir B, Sundfeldt K. Evaluation of ovarian cancer biomarkers HE4 and CA-125 in women presenting with a suspicious cystic ovarian mass. J Gynecol Oncol. 2011;22(4):244–52. 10.3802/jgo.2011.22.4.244 22247801 PMC3254843

[16] Yip P, Chen TH, Seshaiah P, Stephen LL, Michael-Ballard KL, Mapes JP, et al Comprehensive serum profiling for the discovery of epithelial ovarian cancer biomarkers. PLoS One. 2011;6(12):e29533 10.1371/journal.pone.0029533 22216306 PMC3244467

[17] Force UPST. Screening for Ovarian Cancer: US Preventive Services Task Force Recommendation StatementUSPSTF Recommendation: Screening for Ovarian CancerUSPSTF Recommendation: Screening for Ovarian Cancer. JAMA. 2018;319(6):588–94. 10.1001/jama.2017.21926 29450531

[18] Arrieta MC, Stiemsma LT, Amenyogbe N, Brown EM, Finlay B. The intestinal microbiome in early life: health and disease. Frontiers in immunology. 2014;5:427 10.3389/fimmu.2014.00427 25250028 PMC4155789

[19] Spasova DS, Surh CD. Blowing on embers: commensal microbiota and our immune system. Frontiers in immunology. 2014;5:318 10.3389/fimmu.2014.00318 25120539 PMC4112811

[20] Del Chierico F, Vernocchi P, Dallapiccola B, Putignani L. Mediterranean diet and health: food effects on gut microbiota and disease control. International journal of molecular sciences. 2014;15(7):11678–99. 10.3390/ijms150711678 24987952 PMC4139807

[21] Borre YE, Moloney RD, Clarke G, Dinan TG, Cryan JF. The impact of microbiota on brain and behavior: mechanisms & therapeutic potential. Advances in experimental medicine and biology. 2014;817:373–403. 10.1007/978-1-4939-0897-4_17 . 24997043

[22] Petersen C, Round JL. Defining dysbiosis and its influence on host immunity and disease. Cellular microbiology. 2014;16(7):1024–33. 10.1111/cmi.12308 24798552 PMC4143175

[23] Kostic AD, Xavier RJ, Gevers D. The microbiome in inflammatory bowel disease: current status and the future ahead. Gastroenterology. 2014;146(6):1489–99. 10.1053/j.gastro.2014.02.009 24560869 PMC4034132

[24] Mukherjee S, Joardar N, Sengupta S, Sinha Babu SP. Gut microbes as future therapeutics in treating inflammatory and infectious diseases: Lessons from recent findings. The Journal of nutritional biochemistry. 2018;61:111–28. 10.1016/j.jnutbio.2018.07.010 . 30196243 PMC7126101

[25] Rooks MG, Garrett WS. Gut microbiota, metabolites and host immunity. Nature reviews Immunology. 2016;16(6):341–52. 10.1038/nri.2016.42 27231050 PMC5541232

[26] Scher JU, Abramson SB. The microbiome and rheumatoid arthritis. Nature reviews Rheumatology. 2011;7(10):569–78. 10.1038/nrrheum.2011.121 21862983 PMC3275101

[27] Bhatt AP, Redinbo MR, Bultman SJ. The role of the microbiome in cancer development and therapy. CA Cancer J Clin. 2017;67(4):326–44. 10.3322/caac.21398 28481406 PMC5530583

[28] Chase D, Goulder A, Zenhausern F, Monk B, Herbst-Kralovetz M. The vaginal and gastrointestinal microbiomes in gynecologic cancers: a review of applications in etiology, symptoms and treatment. Gynecol Oncol. 2015;138(1):190–200. 10.1016/j.ygyno.2015.04.036 . 25957158

[29] Paul B, Barnes S, Demark-Wahnefried W, Morrow C, Salvador C, Skibola C, et al Influences of diet and the gut microbiome on epigenetic modulation in cancer and other diseases. Clinical epigenetics. 2015;7:112 10.1186/s13148-015-0144-7 26478753 PMC4609101

[30] Molinaro AM, Simon R, Pfeiffer RM. Prediction error estimation: a comparison of resampling methods. Bioinformatics (Oxford, England). 2005;21(15):3301–7. Epub 2005/05/21. 10.1093/bioinformatics/bti499 . 15905277

[31] Hunter VJ, Weinberg JB, Haney AF, Soper JT, Lavin P, Metsch L, et al CA 125 in peritoneal fluid and serum from patients with benign gynecologic conditions and ovarian cancer. Gynecol Oncol. 1990;36(2):161–5. Epub 1990/02/01. 10.1016/0090-8258(90)90165-h . 2404835

[32] Chudecka-Glaz A, Cymbaluk-Ploska A, Menkiszak J, Sompolska-Rzechula A, Byra E, Rzepka-Gorska I. HE4 tumor marker concentration in neoplastic peritoneal effusion and in peritoneal fluid associated with benign gynecological diseases. Journal of ovarian research. 2014;7:22 Epub 2014/02/18. 10.1186/1757-2215-7-22 24528554 PMC3940276

[33] Edgar RC. Search and clustering orders of magnitude faster than BLAST. Bioinformatics. 2010;26(19):2460–1. 10.1093/bioinformatics/btq461 . 20709691

[34] Caporaso JG, Lauber CL, Walters WA, Berg-Lyons D, Huntley J, Fierer N, et al Ultra-high-throughput microbial community analysis on the Illumina HiSeq and MiSeq platforms. The ISME journal. 2012;6(8):1621–4. 10.1038/ismej.2012.8 . 22402401 PMC3400413

[35] McDonald D, Clemente JC, Kuczynski J, Rideout JR, Stombaugh J, Wendel D, et al The Biological Observation Matrix (BIOM) format or: how I learned to stop worrying and love the ome-ome. GigaScience. 2012;1(1):7 10.1186/2047-217X-1-7 23587224 PMC3626512

[36] Oksanen J, Guillaume Blanchet F, Friendly M, Kindt R, Legendre P, et al. Community Ecology Package 2017. p. Ordination methods. Diversity analysis and other functions for community and vegetation ecologists.

[37] McMurdie PJ, Holmes S. Phyloseq: a bioconductor package for handling and analysis of high-throughput phylogenetic sequence data. Pacific Symposium on Biocomputing Pacific Symposium on Biocomputing. 2012:235–46. 22174279 PMC3357092

[38] R Team C. Team RDC.R: A Language And Environment For Statistical Computing. R Foundation for Statistical Computing: Vienna, Austria 2012.

[39] Caporaso JG, Kuczynski J, Stombaugh J, Bittinger K, Bushman FD, Costello EK, et al QIIME allows analysis of high-throughput community sequencing data. Nat Methods. 7 United States2010 p. 335–6. 10.1038/nmeth.f.303 20383131 PMC3156573

[40] Blankenberg D, Von Kuster G, Coraor N, Ananda G, Lazarus R, Mangan M, et al Galaxy: a web-based genome analysis tool for experimentalists. Current protocols in molecular biology. 2010;Chapter 19:Unit 19.0.1–21. Epub 2010/01/14. 10.1002/0471142727.mb1910s89 20069535 PMC4264107

[41] Giardine B, Riemer C, Hardison RC, Burhans R, Elnitski L, Shah P, et al Galaxy: a platform for interactive large-scale genome analysis. Genome research. 2005;15(10):1451–5. 10.1101/gr.4086505 16169926 PMC1240089

[42] Goecks J, Nekrutenko A, Taylor J, Galaxy T. Galaxy: a comprehensive approach for supporting accessible, reproducible, and transparent computational research in the life sciences. Genome biology. 2010;11(8):R86 10.1186/gb-2010-11-8-r86 20738864 PMC2945788

[43] Genuer R, Poggi J-M, Tuleau-Malot C. Variable selection using random forests. Pattern Recognition Letters. 2010;31(14):2225–36. 10.1016/j.patrec.2010.03.014.

[44] Tibshirani R. Regression Shrinkage and Selection Via the Lasso. Journal of the Royal Statistical Society: Series B (Methodological). 1996;58(1):267–88. 10.1111/j.2517-6161.1996.tb02080.x

[45] Szekely GJ, Rizzo ML, Bakirov NK. Measuring and testing dependence by correlation of distances. Ann Statist. 2007;35(6):2769–94. 10.1214/009053607000000505

[46] Mann HB, Whitney DR. On a Test of Whether one of Two Random Variables is Stochastically Larger than the Other. Ann Math Statist. 1947;18(1):50–60. 10.1214/aoms/1177730491

[47] Zhou YH, Gallins P. A Review and Tutorial of Machine Learning Methods for Microbiome Host Trait Prediction. Front Genet. 2019;10:579 Epub 2019/07/12. 10.3389/fgene.2019.00579 31293616 PMC6603228

[48] Kohavi R. A Study of Cross-Validation and Bootstrap for Accuracy Estimation and Model Selection (Abstract). Proceedings of the 14th International Joint Conference on Artificial Intelligence. 1995;2:1137–45.

[49] Pedregosa F, Varoquaux G, Gramfort A, Michel V, Thirion B, Grisel O, et al Scikit-learn: Machine Learning in Python. J Mach Learn Res. 2011;12:2825–30. WOS:000298103200003.

[50] Tibshirani R. Regression shrinkage and selection via the lasso: a retrospective. J R Stat Soc B. 2011;73:273–82. 10.1111/j.1467-9868.2011.00771.x WOS:000290575300001.

[51] Breiman L. Random forests. Mach Learn. 2001;45(1):5–32. 10.1023/A:1010933404324 WOS:000170489900001.

[52] Friedman J, Hastie T, Tibshirani R. Regularization Paths for Generalized Linear Models via Coordinate Descent. Journal of statistical software. 2010;33(1):1–22. 20808728 PMC2929880

[53] Friedman J, Hastie T, Tibshirani R. Regularization Paths for Generalized Linear Models via Coordinate Descent. J Stat Softw. 2010;33(1):1–22. WOS:000275203200001. 20808728 PMC2929880

[54] Liaw A, Wiener M. Classification and Regression by randomForest. R News. 2002;2(3):18–22.

[55] Farolfi A, Gurioli G, Fugazzola P, Burgio SL, Casanova C, Ravaglia G, et al Immune System and DNA Repair Defects in Ovarian Cancer: Implications for Locoregional Approaches. International journal of molecular sciences. 2019;20(10). Epub 2019/05/28. 10.3390/ijms20102569 31130614 PMC6566239

[56] Nakamura M, Bax HJ, Scotto D, Souri EA, Sollie S, Harris RJ, et al Immune mediator expression signatures are associated with improved outcome in ovarian carcinoma. Oncoimmunology. 2019;8(6):e1593811 Epub 2019/05/10. 10.1080/2162402X.2019.1593811 31069161 PMC6492968

[57] Zandi Z, Kashani B, Poursani EM, Bashash D, Kabuli M, Momeny M, et al TLR4 blockade using TAK-242 suppresses ovarian and breast cancer cells invasion through the inhibition of extracellular matrix degradation and epithelial-mesenchymal transition. Eur J Pharmacol. 2019;853:256–63. Epub 2019/04/02. 10.1016/j.ejphar.2019.03.046 . 30930249

[58] Wu YY, Qin YY, Qin JQ, Zhang X, Lin FQ. Diagnostic value of derived neutrophil-to-lymphocyte ratio in patients with ovarian cancer. J Clin Lab Anal. 2019;33(4):e22833 Epub 2019/01/23. 10.1002/jcla.22833 30666724 PMC6528614

[59] Aguilera O, Andres MT, Heath J, Fierro JF, Douglas CW. Evaluation of the antimicrobial effect of lactoferrin on Porphyromonas gingivalis, Prevotella intermedia and Prevotella nigrescens. FEMS Immunol Med Microbiol. 1998;21(1):29–36. Epub 1998/07/10. 10.1111/j.1574-695X.1998.tb01146.x . 9657318

[60] Scher JU, Sczesnak A, Longman RS, Segata N, Ubeda C, Bielski C, et al Expansion of intestinal Prevotella copri correlates with enhanced susceptibility to arthritis. eLife. 2013;2:e01202 Epub 2013/11/07. 10.7554/eLife.01202 24192039 PMC3816614

[61] Lukens JR, Gurung P, Vogel P, Johnson GR, Carter RA, McGoldrick DJ, et al Dietary modulation of the microbiome affects autoinflammatory disease. Nature. 2014;516(7530):246–9. Epub 2014/10/03. 10.1038/nature13788 25274309 PMC4268032

[62] Javurek AB, Spollen WG, Ali AM, Johnson SA, Lubahn DB, Bivens NJ, et al Discovery of a Novel Seminal Fluid Microbiome and Influence of Estrogen Receptor Alpha Genetic Status. Scientific reports. 2016;6:23027 Epub 2016/03/15. 10.1038/srep23027 26971397 PMC4789797

[63] Wu S, Hu R, Nakano H, Chen K, Liu M, He X, et al Modulation of Gut Microbiota by Lonicera caerulea L. Berry Polyphenols in a Mouse Model of Fatty Liver Induced by High Fat Diet. Molecules. 2018;23(12). Epub 2018/12/20. 10.3390/molecules23123213 30563142 PMC6321169

[64] Goker M, Gronow S, Zeytun A, Nolan M, Lucas S, Lapidus A, et al Complete genome sequence of Odoribacter splanchnicus type strain (1651/6). Stand Genomic Sci. 2011;4(2):200–9. Epub 2011/06/17. 10.4056/sigs.1714269 21677857 PMC3111987

[65] Gomez-Arango LF, Barrett HL, McIntyre HD, Callaway LK, Morrison M, Dekker Nitert M, et al Increased Systolic and Diastolic Blood Pressure Is Associated With Altered Gut Microbiota Composition and Butyrate Production in Early Pregnancy. Hypertension. 2016;68(4):974–81. Epub 2016/08/17. 10.1161/HYPERTENSIONAHA.116.07910 . 27528065

[66] Golob JL, Pergam SA, Srinivasan S, Fiedler TL, Liu C, Garcia K, et al Stool Microbiota at Neutrophil Recovery Is Predictive for Severe Acute Graft vs Host Disease After Hematopoietic Cell Transplantation. Clinical infectious diseases: an official publication of the Infectious Diseases Society of America. 2017;65(12):1984–91. Epub 2017/10/12. 10.1093/cid/cix699 29020185 PMC5850019

[67] Schnabl B, Brenner DA. Interactions between the intestinal microbiome and liver diseases. Gastroenterology. 2014;146(6):1513–24. Epub 2014/01/21. 10.1053/j.gastro.2014.01.020 24440671 PMC3996054

[68] Machiels K, Joossens M, Sabino J, De Preter V, Arijs I, Eeckhaut V, et al A decrease of the butyrate-producing species Roseburia hominis and Faecalibacterium prausnitzii defines dysbiosis in patients with ulcerative colitis. Gut. 2014;63(8):1275–83. Epub 2013/09/12. 10.1136/gutjnl-2013-304833 . 24021287

[69] Naftali T, Reshef L, Kovacs A, Porat R, Amir I, Konikoff FM, et al Distinct Microbiotas are Associated with Ileum-Restricted and Colon-Involving Crohn's Disease. Inflammatory bowel diseases. 2016;22(2):293–302. Epub 2016/01/12. 10.1097/MIB.0000000000000662 . 26752462

[70] Kaakoush NO. Insights into the Role of Erysipelotrichaceae in the Human Host. Frontiers in cellular and infection microbiology. 2015;5:84 Epub 2015/12/05. 10.3389/fcimb.2015.00084 26636046 PMC4653637

[71] Africa CW, Nel J, Stemmet M. Anaerobes and bacterial vaginosis in pregnancy: virulence factors contributing to vaginal colonisation. Int J Environ Res Public Health. 2014;11(7):6979–7000. Epub 2014/07/12. 10.3390/ijerph110706979 25014248 PMC4113856

[72] Bhattarai Y, Muniz Pedrogo DA, Kashyap PC. Irritable bowel syndrome: a gut microbiota-related disorder? Am J Physiol Gastrointest Liver Physiol. 2017;312(1):G52–G62. Epub 2016/11/25. 10.1152/ajpgi.00338.2016 27881403 PMC5283907

[73] Gregory JC, Buffa JA, Org E, Wang Z, Levison BS, Zhu W, et al Transmission of atherosclerosis susceptibility with gut microbial transplantation. J Biol Chem. 2015;290(9):5647–60. Epub 2015/01/01. 10.1074/jbc.M114.618249 25550161 PMC4342477

[74] Duarte SMB, Stefano JT, Miele L, Ponziani FR, Souza-Basqueira M, Okada L, et al Gut microbiome composition in lean patients with NASH is associated with liver damage independent of caloric intake: A prospective pilot study. Nutrition, metabolism, and cardiovascular diseases: NMCD. 2018;28(4):369–84. Epub 2018/02/28. 10.1016/j.numecd.2017.10.014 . 29482963

[75] Gupta RS, Mok A. Phylogenomics and signature proteins for the alpha proteobacteria and its main groups. BMC Microbiol. 2007;7:106 Epub 2007/11/30. 10.1186/1471-2180-7-106 18045498 PMC2241609

[76] van Passel MW, Kant R, Zoetendal EG, Plugge CM, Derrien M, Malfatti SA, et al The genome of Akkermansia muciniphila, a dedicated intestinal mucin degrader, and its use in exploring intestinal metagenomes. PLoS One. 2011;6(3):e16876 Epub 2011/03/11. 10.1371/journal.pone.0016876 21390229 PMC3048395

[77] Routy B, Le Chatelier E, Derosa L, Duong CPM, Alou MT, Daillere R, et al Gut microbiome influences efficacy of PD-1-based immunotherapy against epithelial tumors. Science. 2018;359(6371):91–7. Epub 2017/11/04. 10.1126/science.aan3706 . 29097494

[78] Bai J, Behera M, Bruner DW. The gut microbiome, symptoms, and targeted interventions in children with cancer: a systematic review. Supportive care in cancer: official journal of the Multinational Association of Supportive Care in Cancer. 2018;26(2):427–39. Epub 2017/11/24. 10.1007/s00520-017-3982-3 . 29168036

[79] Mert I, Walther-Antonio M, Mariani A. Case for a role of the microbiome in gynecologic cancers: Clinician's perspective. The journal of obstetrics and gynaecology research. 2018;44(9):1693–704. 10.1111/jog.13701 . 30069974

[80] Burns MB, Montassier E, Abrahante J, Priya S, Niccum DE, Khoruts A, et al Colorectal cancer mutational profiles correlate with defined microbial communities in the tumor microenvironment. PLoS genetics. 2018;14(6):e1007376 10.1371/journal.pgen.1007376 29924794 PMC6028121

[81] Champer M, Wong AM, Champer J, Brito IL, Messer PW, Hou JY, et al The role of the vaginal microbiome in gynaecological cancer. BJOG: an international journal of obstetrics and gynaecology. 2018;125(3):309–15. 10.1111/1471-0528.14631 . 28278350

[82] Roy S, Trinchieri G. Microbiota: a key orchestrator of cancer therapy. Nat Rev Cancer. 2017;17(5):271–85. Epub 2017/03/18. 10.1038/nrc.2017.13 . 28303904

[83] Shah MS, DeSantis TZ, Weinmaier T, McMurdie PJ, Cope JL, Altrichter A, et al Leveraging sequence-based faecal microbial community survey data to identify a composite biomarker for colorectal cancer. Gut. 2018;67(5):882–91. Epub 2017/03/28. 10.1136/gutjnl-2016-313189 . 28341746

[84] Ai L, Tian H, Chen Z, Chen H, Xu J, Fang JY. Systematic evaluation of supervised classifiers for fecal microbiota-based prediction of colorectal cancer. Oncotarget. 2017;8(6):9546–56. Epub 2017/01/07. 10.18632/oncotarget.14488 28061434 PMC5354752

[85] Fowler JR. Culdocentesis: UpToDate, Inc.; 2019 [updated 08/29/2019]. Available from: https://www.uptodate.com/contents/culdocentesis.

[86] Grillo D, Stienmier RH, Lowell DM. Early diagnosis of ovarian carcinoma by culdocentesis. Obstet Gynecol. 1966;28(3):346–50. Epub 1966/09/01. . 5917741

[87] Jamain B, Letessier A, Cathely JP. [10 years' experience with surgery of Douglas' pouch]. Gynecol Obstet (Paris). 1969;68(1):73–8. Epub 1969/01/01. . 5404675

[88] Chen PC, Sickler GK, Dubinsky TJ, Maklad N, Jacobi RL, Weaver JE. Sonographic detection of echogenic fluid and correlation with culdocentesis in the evaluation of ectopic pregnancy. AJR American journal of roentgenology. 1998;170(5):1299–302. Epub 1998/05/09. 10.2214/ajr.170.5.9574606 . 9574606

[89] Roberts MR, Jackimczyk K, Marx J, Rosen P. Diagnosis of ruptured ectopic pregnancy with peritoneal lavage. Annals of emergency medicine. 1982;11(10):556–8. Epub 1982/10/01. 10.1016/s0196-0644(82)80431-9 . 7125318

[90] Romero R, Copel JA, Kadar N, Jeanty P, Decherney A, Hobbins JC. Value of culdocentesis in the diagnosis of ectopic pregnancy. Obstet Gynecol. 1985;65(4):519–22. Epub 1985/04/01. . 3982726

[91] Bellia A, Vitale SG, Lagana AS, Cannone F, Houvenaeghel G, Rua S, et al Feasibility and surgical outcomes of conventional and robot-assisted laparoscopy for early-stage ovarian cancer: a retrospective, multicenter analysis. Arch Gynecol Obstet. 2016;294(3):615–22. Epub 2016/04/05. 10.1007/s00404-016-4087-9 . 27040423

[92] Minig L, Padilla Iserte P, Zorrero C, Zanagnolo V. Robotic Surgery in Women With Ovarian Cancer: Surgical Technique and Evidence of Clinical Outcomes. J Minim Invasive Gynecol. 2016;23(3):309–16. Epub 2015/11/06. 10.1016/j.jmig.2015.10.014 . 26538410

